# Successful application of the snare technique for the deployment of the proximal portion of a Zenith Alpha Thoracic Endovascular Graft: a case report

**DOI:** 10.1186/s42155-020-00164-9

**Published:** 2020-10-01

**Authors:** Hiroki Nagayama, Tetsuhiro Otsuka, Atsushi Miyazaki, Shunsuke Taguchi, Seiji Matsukuma, Tsuneo Ariyoshi

**Affiliations:** 1 Department of Radiology, National Hospital Organization Nagasaki Medical Center, 2-1001-1 Kubara, Omura, Nagasaki Japan; 2 Department of Cardiovascular Surgery, National Hospital Organization Nagasaki Medical Center, Omura, Japan

**Keywords:** Thoracic endovascular aortic repair, Incomplete deployment, Bare stent, Proximal portion of the graft, Snare, Endovascular

## Abstract

**Background:**

Open surgery can be required or even fatal if incomplete deployment of stent graft (SG) occurs. We herein report the first case in which a snare was successfully used to perform endovascular therapeutic troubleshooting on the proximal portion of a Zenith Alpha thoracic endovascular graft proximal component that showed incomplete deployment.

**Case presentation:**

The patient was an 80-year-old woman. She underwent thoracic endovascular aortic repair (TEVAR) for subacute phase Stanford type B ulcer-like projection aortic dissection. Although the ulcer-like projection disappeared, a follow-up computed tomography angiogram (CTA) obtained approximately 1 year postoperatively showed type Ia and Ib endoleaks. Since there is a high risk of rupture as the aneurysm diameter increases, we determined that an additional SG was indicated. An attempt was made to place the SG in Zone 3, but as the lesser curvature side of the proximal portion stopped in a position that was perpendicular to the vascular wall (downward facing), the SG proximal portion did not completely expand. A guiding sheath was inserted into the aortic arch via the left brachial artery, and, using a snare that we inserted via the femoral artery, we grasped the guiding sheath. A catheter and guidewire (GW) were inserted via the guiding sheath and then rotated under the lesser curvature of the SG proximal portion; the GW was then passed through the loop of the snare. This allowed us to insert the hard loop structure under the SG proximal portion, which in turn allowed successful repair of the incomplete deployment of the SG. Type Ia and Ib endoleaks remained but were less than those before additional TEVAR.

One week later, she was discharged. One year later, CT showed no interval change in the size of aortic aneurysm with dissection, and she has been followed on an outpatient basis.

**Conclusions:**

When the endovascular diameter of the proximal aortic arch is large, incomplete deployment of the proximal portion of a Zenith Alpha thoracic endovascular graft can occur, but bailout is possible through the use of the snare technique as endovascular therapy.

## Background

The Zenith Alpha thoracic endovascular graft (Cook Medical, Bloomington, IN, USA) is a low-profile system that facilitates the deployment of its proximal portion (Illig et al. 2015; Torsello et al. 2012) and could be a viable alternative for treating patients with an aortic arch proximal landing zone, to facilitate precise deployment (Pane et al. 2018).

This is the first report of the successful use of a snare to troubleshoot the deployment failure of the proximal portion of a Zenith Alpha thoracic endovascular graft.

## Case presentation

An 80-year-old woman who underwent thoracic endovascular aortic repair (TEVAR) for subacute phase Stanford type B ulcer-like projection aortic dissection revealed type Ia and Ib endoleaks (ELs) on a 1-year follow-up computed tomography angiogram (CTA). We determined that additional stent grafting was indicated.

Preoperative CTA showed that the proximal neck diameter was between 40 and 43 mm; therefore, during the initial TEVAR, two stent grafts (SGs) were utilized. Under general anesthesia, 8-F, 5-F, and 6-F sheaths were inserted via the left common femoral artery, right femoral artery, and left brachial artery, respectively.

The type Ib EL, located in the distal descending thoracic aorta, was identified using digital subtraction angiography (DSA). A 0.035-in. Lunderquist (Cook Medical, Bloomington, IN, USA) guidewire (GW) was inserted via the left common femoral artery. Thereafter, a Zenith Alpha proximal component (diameter/length: 46/233 mm) with an introduction system diameter of 20-F was inserted. It was placed approximately 15 mm from the SG inferior component (Th12 level), implanted previously.

**Fig. 1:**
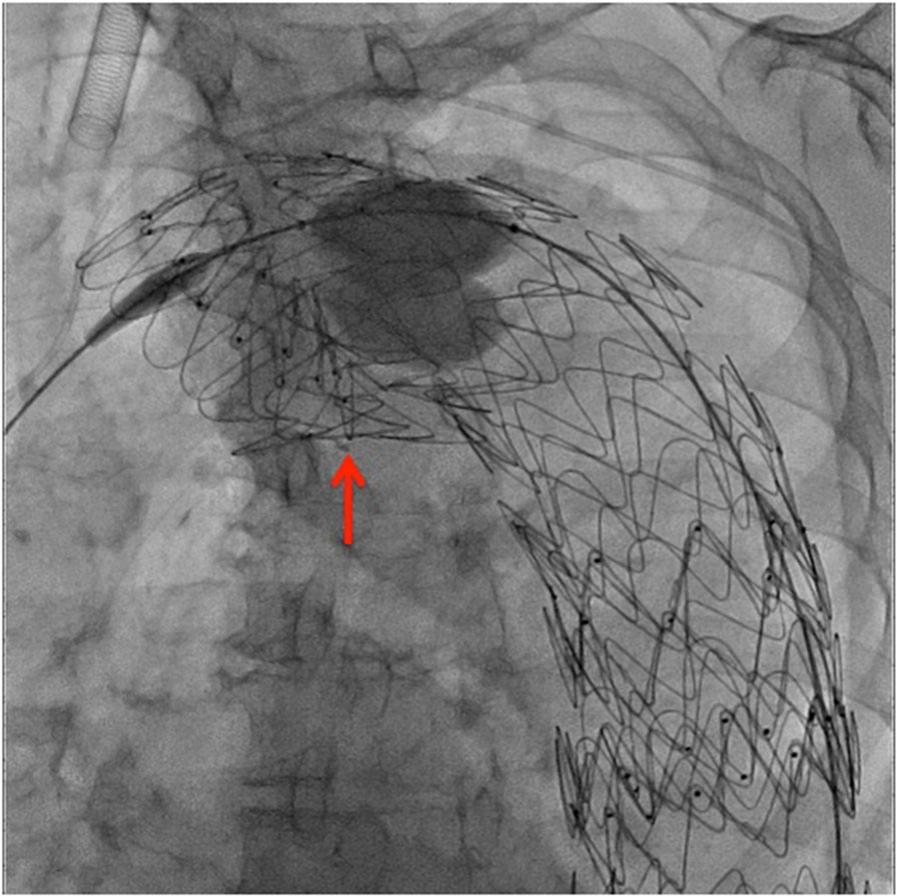
Fluoroscopic image immediately after additional stent grafting. The inner curved side of the proximal portion of the additional SG stopped perpendicular to the vascular wall (red arrow) and failed to deploy properly

**Fig. 2:**
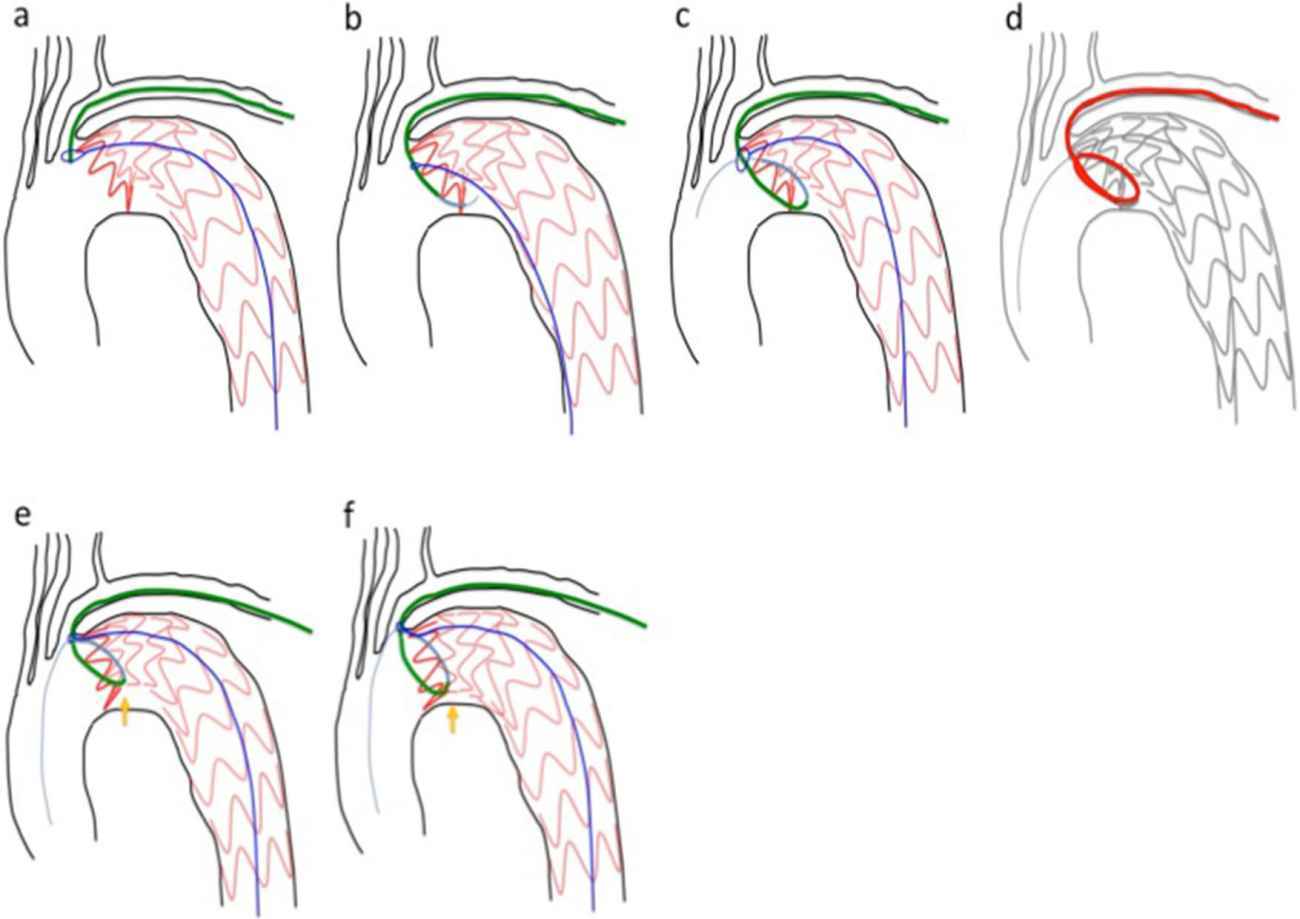
Schema of the double-snare technique. **a** Snare grasping of the distal side of the guiding sheath via left brachial artery. **b** Pulling the snare wire and advancing the guiding sheath toward the inner curved side of the proximal portion of the SG. **c** Passing the Radifocus GW through the temporarily opened snare loop. **d** The hard loop structure (guiding sheath end/IMA catheter/GW) under the inner curved side of the proximal portion of the SG. **e**, **f** Pulling the guiding sheath toward the left subclavian artery and repositioning (yellow arrow) the inner curved side of the proximal portion of the SG

The type Ia EL was identified in the aortic arch, and a Zenith Alpha proximal component (diameter/length: 46/179 mm) with an introduction system diameter of 20-F was inserted. An attempt was made to deploy the SG directly posterior to the bifurcation of the left subclavian artery in Zone 3 (approximately 15 mm from the previously placed SG’s upper end); however, the inner curved side of the proximal portion stopped perpendicular to the vascular wall and failed to deploy properly (Fig. 1). Various unsuccessful attempts at repositioning were performed. Finally, the sheath in the left brachial artery was replaced with a 6-F guiding sheath (length: 53 cm), which was inserted into the aortic arch. The right femoral artery sheath was replaced with a 6-F sheath, and an Amplatz Goose Neck snare (Medtronic, Minneapolis, MN, USA) (wire diameter: 25 mm) was inserted through it and used to grasp the distal side of the abovementioned guiding sheath (Fig. 2a). The end of the left brachial artery sheath was rotated toward the inner curved side of the SG proximal component. Using a 4-F shepherd hook catheter, we passed a 0.035-in. Radifocus (Terumo Medical Corporation, Tokyo, Japan) GW under the inner curved side of the SG proximal component.

**Fig. 3:**
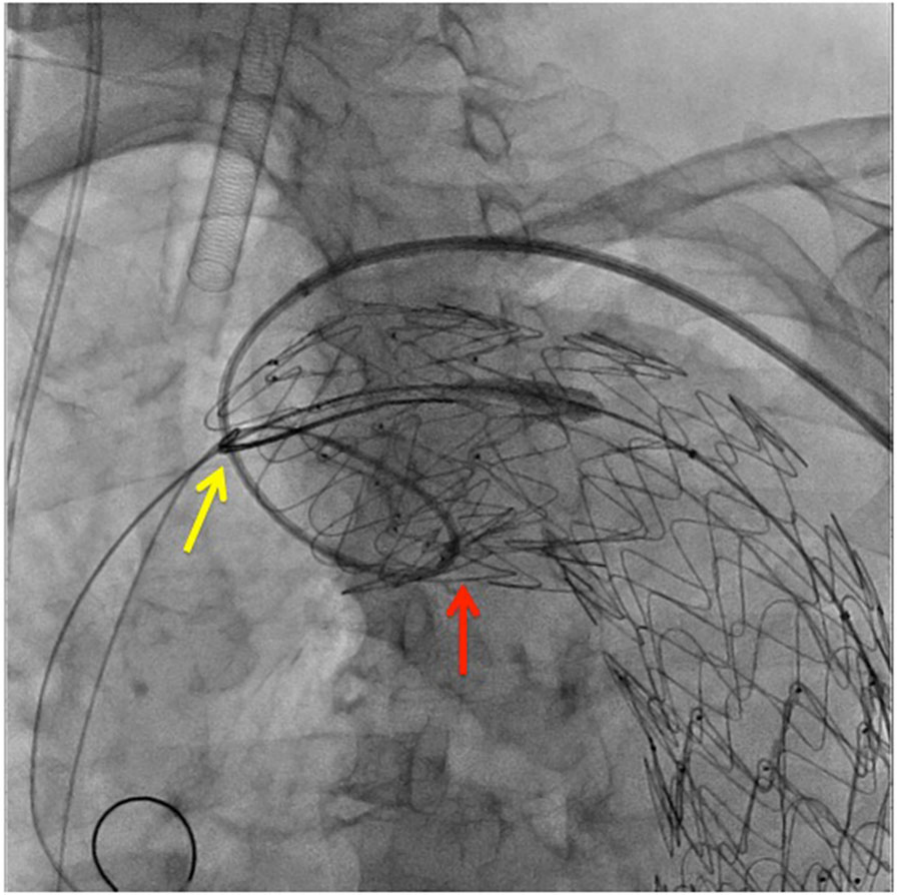
Fluoroscopic image immediately after repair of the incomplete deployment of the SG using the double-snare technique. A catheter and GW are inserted through a guiding sheath and rotated under the stent-graft. The GW is passed through the loop (yellow arrow) of a snare, permitting deployment of the SG (red arrow)

We then pulled the snare wire forward, to the greatest extent possible, and by placing tension on it, faced the left brachial artery sheath toward the lesser curved side of the proximal portion of the SG (Fig. 2b); we used the 0.035-in. Radifocus GW, to insert a 6-F internal mammary artery (IMA) catheter as deep as possible into the insufficiently deployed portion. After passing through and rotating it, we passed the Radifocus GW through the temporarily opened snare loop, and by grasping it (Fig. 2c), we could pass the hard loop structure (guiding sheath end/IMA catheter/GW) under the inner curved side of the SG proximal portion (Fig. 2d). We then carefully and slowly pulled the guiding sheath to its original position, which repositioned the inner curved side of the SG proximal portion to a position parallel to the vascular wall (Fig. 2e, f), where it was successfully deployed (Fig. 3).

**Fig. 4:**
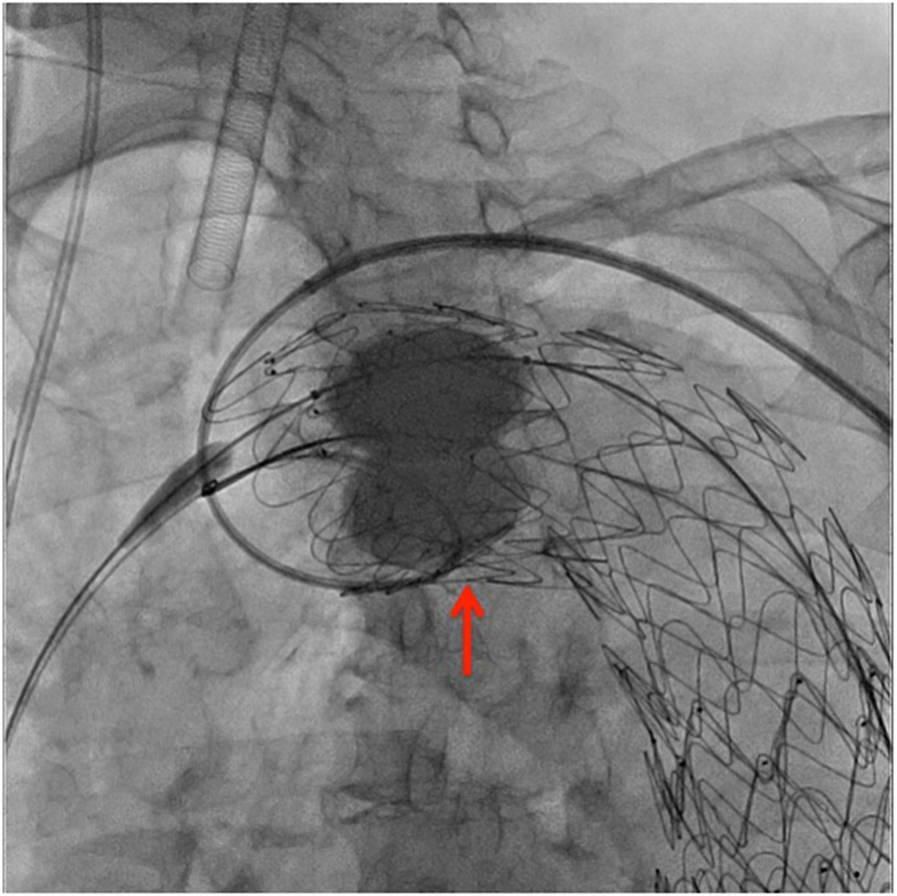
Fluoroscopic image under reinforcement of proximal sealing using ballooning. Pressure applied on the proximal side of the SG using Tri-Lobe Balloon Catheter (red arrow), which reinforces the proximal sealing

We opened the snare loop and used a Tri-Lobe Balloon Catheter (W. L. GORE & Associates, Flagstaff, AZ, USA) to apply pressure from the lumen side of the SG, which allowed us to remove the 6-F IMA catheter. Once again, we applied pressure on the SG proximal side using the Tri-Lobe Balloon Catheter, reinforced the proximal sealing (Fig. 4), and performed DSA. Type Ia and Ib ELs remained but were less than those before additional TEVAR. One week later, she was discharged. One year later, CT showed no interval change in size of aortic aneurysm with dissection, and she has been followed on an outpatient basis.

## Conclusions

The proximal portion of the Zenith Alpha thoracic endovascular graft proximal component has a mechanism that reduces bird beak formations proximal to the aortic arch to a great extent. However, based on our experience, if the diameter of the proximal blood vessel is 40 mm or larger, incomplete deployment is a possibility when placing the SG in a region from the proximal aortic arch to the isthmus.

In the present case, the incomplete deployment might have been caused by interference with the previously placed proximal bare stent portion of SG. When placing additional SGs following TEVAR, caution must be exercised to prevent the bare stents from interfering with each other, particularly when the Zenith Alpha thoracic endovascular graft is used.

Non-deployment of the proximal portion of a Zenith Alpha thoracic endovascular graft could be rectified using the double-snare technique described here, in which a snare wire is passed through the left subclavian and femoral artery for Zone 3 placement.

## Data Availability

The datasets during and/or analyzed during the current case report are available from the corresponding author on reasonable request.

## Abbreviations

CTA: Computed tomography angiogram
DSA: Digital subtraction angiography
GW: Guidewire
IMA: Internal mammary artery
SG: Stentgraft
TEVAR: Thoracic endovascular aortic repair
